# Neighborhood Socioeconomic Deprivation and 30-Day Outcomes After Admission for Common Gastrointestinal Conditions: A Large Nationwide Study

**DOI:** 10.1016/j.gastha.2025.100614

**Published:** 2025-01-09

**Authors:** Beau Blass, Jay B. Lusk, Hannah Mahoney, Molly N. Hoffman, Amy G. Clark, Jonathan Bae, Matthew J. Townsend, Amit Patel, Andrew J. Muir, Bradley G. Hammill

**Affiliations:** 1 Duke University School of Medicine, Durham, North Carolina; 2 Department of Medicine, Duke University School of Medicine, Durham, North Carolina; 3 Department of Population Health Sciences, Duke University School of Medicine, Durham, North Carolina; 4 Durham Veterans Affairs Medical Center, Durham, North Carolina

**Keywords:** Neighborhood Deprivation, Gastrointestinal Disease, Health Disparities, Area Deprivation Index, Equity, Socioeconomic

## Abstract

**Background and Aims:**

To study the associations of neighborhood socioeconomic disadvantage with 30-day mortality and readmission for common gastrointestinal conditions, adjusting for individual demographics, comorbidities, access to health-care resources, and treatment facility characteristics.

**Methods:**

We analyzed a nationwide sample of United States Medicare beneficiaries hospitalized from 2017 to 2019 for common gastrointestinal diseases, grouped by diagnosis-related groups. We then estimated the association of neighborhood socioeconomic disadvantage, measured by the Area Deprivation Index, with 30-day mortality and readmission utilizing logistic regression models with restricted cubic splines. We performed multistep adjustments for individual socioeconomic status and demographics, medical comorbidities, access to inpatient and outpatient health-care resources, and hospital-level characteristics.

**Results:**

In total, 1,293,483 patients in the mortality cohort and 1,289,106 patients in the readmission cohort were included in analysis. The fully adjusted model demonstrated an association between neighborhood deprivation and 30-day mortality for patients with common gastrointestinal diseases, with the strongest associations for nonmalignant pancreatic disorders (odds ratio [OR] 1.59, 95% confidence interval [CI] 1.25–2.01), esophageal disorders (OR 1.50, 95% 1.02–2.21), gastrointestinal hemorrhage (OR 1.40, 95% CI 1.29–1.52), and biliary tract disorders (OR 1.40, 95% CI 1.16–1.69) in the most deprived groups. Neighborhood deprivation was not associated with 30-day readmission after full adjustment.

**Conclusion:**

We describe an independent association between neighborhood deprivation and 30-day mortality for patients with common gastrointestinal diseases, which remains even after controlling for individual poverty, demographics and comorbidities, access to health-care resources, and characteristics of treating facilities.

## Introduction

Neighborhood socioeconomic disadvantage is increasingly recognized as a significant contributor to health disparities. Neighborhood disadvantage encapsulates many social, structural, and environmental forces that collectively influence individual health, including limited access to health-care facilities, increased economic stress, environmental pollution, poor housing, inadequate infrastructure, and paucities of reliable transportation and safe outdoor spaces.^1^, ^2^, ^3^, ^4^ In fact, neighborhood disadvantage independently predicts certain poor health outcomes, including overall mortality, even after controlling for individual socioeconomic status (SES).^1^^,^^2^^,^^5^^,^^6^

Socioeconomic disadvantage at both individual and neighborhood levels has been associated with differences in treatment and outcomes for patients with gastrointestinal diseases. Single-institution studies have shown that deprivation at the neighborhood level is associated with failure to be waitlisted and death during liver transplant evaluation, and reduced initiation of antiviral therapy for patients with hepatitis C infection.^7^^,^^8^ At a national level, individual poverty has been associated with increased mortality among patients with nonalcoholic fatty liver disease,^9^ and higher household zip-code income has been associated with lower in-hospital mortality for patients with decompensated cirrhosis.^10^ However, no study has evaluated the effects of neighborhood-level deprivation on population-level outcomes in a nationwide cohort of patients with common gastrointestinal diseases.

Because the relationships between neighborhood socioeconomic deprivation and gastrointestinal disease outcomes may be confounded by significant overlapping factors such as individual SES and demographics, medical comorbidities, access to inpatient and outpatient health-care resources, and hospital-level characteristics, ascertaining whether neighborhood deprivation is independently associated with mortality and readmission warrants acknowledging and controlling for these factors. Therefore, we sought to characterize the associations of neighborhood socioeconomic disadvantage on 30-day mortality and readmission for patients admitted with common gastrointestinal conditions in a large, nationally representative cohort of Medicare patients using a sequential adjustment strategy. Identification of associations between neighborhood deprivation and outcomes following admission for common gastrointestinal conditions may help guide interventions at the national, municipal, and institutional level(s) focused on root disparities.

## Materials and Methods

### Data Source

Data were accessed from the Centers for Medicare and Medicaid Services (CMS) Virtual Research Data Center and included all fee-for-service Medicare claims from 2017 to 2019, including inpatient, outpatient, and carrier claims. Data from 2016 were used as a look-back to ascertain comorbidities; accordingly, the first eligible index date was January 1, 2017.

### Study Cohorts

Our case selection strategy was modeled after the CMS methodology for calculating mortality and readmission quality metrics to provide maximal relevance for health policy.^11^^,^^12^ Patients were included in 1 of 2 cohorts—a mortality cohort or a readmission cohort. Inclusion criteria for both cohorts included age ≥65 years, continuous enrollment in fee-for-service Medicare for ≥1 month (or until death) following the index admission, and 1 year of prior fee-for-service Medicare enrollment preceding the index admission (for comorbidity ascertainment). For the mortality cohort, hospitalizations were excluded if the patient was transferred to or from another acute care hospital. For the readmission cohort, hospitalizations were excluded if the patient left the hospital against medical advice, died in-hospital, was transferred to another acute-care hospital, or was hospitalized within 30 days of discharge from another hospitalization in the same category. In each cohort, a random, single admission per study group, defined below, was selected as the index admission if the patient was hospitalized multiple times during the study period, consistent with CMS methodology.^11^

### Exposures and Outcomes

The primary exposure was neighborhood socioeconomic deprivation, as indicated by the Area Deprivation Index (ADI).^13^ The ADI is a summary score generated at the level of census block groups from multiple socioeconomic indicators such as income, employment, access to key infrastructure, housing characteristics, and educational attainment. The ADI is reported as a national percentile from 1 (least deprivation) to 100 (most deprivation). We used ZIP +4 codes to link patients to the census block group corresponding to their area of residence.^14^ Our primary outcomes were all-cause mortality within 30 days of admission, and unplanned readmission within 30 days of discharge, where planned and unplanned readmissions were distinguished according to CMS methodology.^12^

### Study Groups and Comorbidity Ascertainment

Patient demographic information, along with enrollment and, if applicable, mortality dates, were obtained from the Medicare Beneficiary Summary File. We used diagnosis-related groups (DRGs), which identify the primary reason why a patient was hospitalized, to categorize admissions within 10 groups as follows: gastrointestinal hemorrhage (DRG 377–379), gastroenteritis and esophagitis (DRG 319–392), gastrointestinal obstruction (DRG 388–390), appendicitis and peritoneal infections (DRG 371–373), biliary tract disorders (DRG 444–446), disorders of the pancreas except malignancy (DRG 438–440), liver disease (DRG 432–434 and DRG 441–443), peptic ulcer disease (DRG 380–382 and 383–384), inflammatory bowel disease (DRG 385–387), and esophageal disorders (DRG 368–370). Comorbidities were ascertained using a 1-year lookback in both inpatient and outpatient claims, using validated algorithms.^15^^,^^16^

### Statistical Analysis

The distribution of each study group was described using percentages and frequencies for categorical variables and means with standard deviations for continuous variables. We grouped patients by ADI into low (ADI 86–100), middle (ADI 16–85), and high (ADI 1–15) neighborhood SES. These cutoffs were chosen based on previous work that demonstrated close association between the 15% threshold and Medicare rehospitalization rates.^17^ We then estimated unadjusted logistic regression models using generalized estimating equations to account for patient clustering within hospitals, and generated odds ratios (ORs) with 95% confidence intervals (CIs). To account for the potential of ADI to vary nonmonotonically with outcomes across the ADI score range, we employed restricted cubic splines with 4 knots (at ADI scores of 5, 30, 70, and 95).^18^ We reported estimates at specific values of ADI and produced figures showing the relationships between ADI, as a continuous variable, and outcomes. This approach allows for more precise modeling of potentially nonlinear relationships. For completeness, we reported in supplemental tables the associations between ADI, as a categorical variable, and both outcomes. We utilized a multistep adjustment strategy where we first adjusted for age, race and ethnicity, sex, individual SES (using dual-eligibility for Medicare and Medicaid as a surrogate measure), end-stage renal disease status (as some patients independently qualify for Medicare due to this reason), and the 29 Elixhauser comorbid medical conditions.^15^ We adjusted for individual Elixhauser comorbid medical conditions rather than using a summary score. We then additionally adjusted for county-level access to health-care resources, specifically number of primary care providers per capita, number of hospital beds per capita, and specialist physicians per capita, derived from the 2019 Area Health Resources File obtained from the Health Resources and Services Administration.^19^ We defined residence in a rural area according to the Rural Urban Continuum Codes in the Area Regional Health File, where values of 1–3 were categorized as urban and 4–9 were categorized as rural. Finally, we adjusted for the characteristics of the admitting hospitals, specifically ownership status (private vs public), teaching status, and number of beds.

Statistical analyses were performed using SAS v9.4 (SAS Institute, Cary, NC). This study was approved by the Duke University Institutional Review Board, protocol number Pro00106448.

## Results

In total, 1,293,483 patients met the inclusion criteria for the mortality cohort and 1,289,106 patients for the readmission cohort; patients could be included in both cohorts. Within the mortality cohort, 31% were hospitalized for gastrointestinal hemorrhage, 27% for gastroenteritis and esophagitis, 13% for gastrointestinal obstruction, 7% for appendicitis and peritoneal infections, 6% for biliary tract disorders, 5% for disorders of pancreas except malignancy, 5% for liver disease, 3% for peptic ulcer disease, 1% for inflammatory bowel disease, and 1% for esophageal disorders. A similar distribution of demographics and admission diagnoses was observed in the readmission cohort.

Demographic characteristics, access to health-care resources, and observed outcomes for the mortality and readmission cohorts, stratified by admission group, are shown in Tables 1 and 2. These data are also stratified by neighborhood SES in Tables A1 and 2. Within the mortality cohort, compared to patients from high (ADI 1–15) and middle neighborhood SES (ADI 16–85) groups, patients from low-SES neighborhoods (ADI 86–100) were younger, more likely to be female, more often dually eligible for Medicare–Medicaid, more likely to identify as Black or Hispanic, and were more likely to reside in a rural area. Patients in the low-neighborhood-SES group also experienced greater crude 30-day mortality (7.3% vs 6.9% for ADI 16%–85% and 6.2% for ADI 1–15). Similar demographic trends and disparities in 30-day outcomes were observed in the readmission cohort. These outcomes are stratified by neighborhood SES and displayed by DRG in Table 3. Differences in 30-day mortality by neighborhood SES group were statistically significant for all assessed DRGs except for esophageal disorders, inflammatory bowel disease, and peptic ulcer disease.

**Table 1:** Demographics, 30-Day Outcomes, Access to Health-Care Resources, and Characteristics of Admitting Health-Care Facilities Stratified by Admission Groups Within the Mortality Cohort

Variable	Gastrointestinal hemorrhage (N = 403,484)	Disorders of pancreas except malignancy (N = 70,129)	Liver disease (N = 64,373)	Esophageal disorders (N = 12,435)	Appendicitis and peritoneal infections (N = 92,994)	Gastrointestinal obstruction (N = 171,895)	Gastroenteritis and esophagitis (N = 351,035)	Inflammatory bowel disease (N = 18,229)	Peptic ulcer disease (N = 36,059)	Biliary tract disorders (N = 72,850)
Main outcome										
Observed mortality	29,996 (7.4)	3126 (4.5)	13,874 (21.6)	1138 (9.2)	6814 (7.3)	11,173 (6.5)	13,907 (4.0)	646 (3.5)	2747 (7.6)	4998 (6.9)
Demographics										
Age (y), mean (SD)	79.8 (8.3)	76.5 (8.1)	74.1 (7.0)	77.3 (8.1)	78.5 (8.3)	78.5 (8.3)	78.2 (8.3)	76.1 (7.5)	78.2 (8.2)	79.4 (8.6)
Legal sex, female	217,519 (53.9)	38,367 (54.7)	30,671 (47.6)	6342 (51.0)	59,219 (63.7)	99,767 (58.0)	237,420 (67.6)	11,569 (63.5)	19,898 (55.2)	40,085 (55.0)
Race/ethnicity										
Asian	6824 (1.7)	1222 (1.7)	1202 (1.9)	245 (2.0)	1037 (1.1)	2500 (1.5)	4806 (1.4)	177 (1.0)	647 (1.8)	1735 (2.4)
Black	45,831 (11.4)	6300 (9.0)	4433 (6.9)	1329 (10.7)	6198 (6.7)	14,814 (8.6)	30,311 (8.6)	1088 (6.0)	3077 (8.5)	5331 (7.3)
Hispanic	6124 (1.5)	1365 (1.9)	2033 (3.2)	243 (2.0)	1379 (1.5)	2691 (1.6)	6391 (1.8)	195 (1.1)	646 (1.8)	1694 (2.3)
Other/Unknown	10,573 (2.6)	2213 (3.2)	2664 (4.1)	410 (3.3)	2264 (2.4)	4759 (2.8)	8454 (2.4)	480 (2.6)	949 (2.6)	2369 (3.3)
White	334,132 (82.8)	59,029 (84.2)	54,041 (83.9)	10,208 (82.1)	82,116 (88.3)	147,131 (85.6)	301,073 (85.8)	16,289 (89.4)	30,740 (85.2)	61,721 (84.7)
Dual Medicare/Medicaid eligible	87,006 (21.6)	13,790 (19.7)	16,132 (25.1)	2862 (23.0)	19,023 (20.5)	33,164 (19.3)	74,624 (21.3)	2914 (16.0)	8500 (23.6)	15,745 (21.6)
Medical history										
Elixhauser mortality index, mean (SD)	19.2 (16.0)	15.6 (14.5)	30.3 (15.9)	23.0 (16.3)	24.1 (15.3)	17.4 (15.1)	17.7 (14.8)	16.5 (14.0)	20.0 (15.9)	19.3 (15.7)
Regional information										
Rural area	75,406 (18.7)	15,779 (22.5)	12,393 (19.3)	2188 (17.6)	19,375 (20.8)	36,198 (21.1)	68,950 (19.6)	3103 (17.0)	6090 (16.9)	13,729 (18.8)
Primary care providers per 100,000 persons, mean (SD)	74.7 (32.4)	72.3 (32.3)	73.1 (32.3)	75.1 (32.2)	73.6 (32.5)	74.1 (32.2)	74.2 (32.5)	75.6 (32.2)	75.0 (31.8)	75.6 (32.7)
Total specialists per 100,000 persons, mean (SD)	222.6 (182.1)	205.8 (174.3)	213.9 (178.2)	225.3 (178.0)	214.2 (180.0)	215.0 (178.3)	219.7 (181.4)	225.8 (180.8)	222.1 (174.7)	228.1 (188.7)
Hospital beds per 10,000 persons, mean (SD)	28.9 (22.2)	28.4 (23.3)	28.3 (22.1)	28.6 (19.7)	28.6 (21.8)	28.6 (22.7)	29.0 (22.9)	28.3 (21.6)	28.5 (21.0)	28.6 (21.8)
Distance to the closest hospital, miles, mean (SD)	4.1 (5.2)	4.3 (5.3)	4.3 (5.4)	4.2 (5.2)	4.2 (5.2)	4.1 (5.1)	4.1 (5.1)	4.1 (5.0)	4.1 (5.5)	4.1 (5.2)
Hospital information										
Number of beds, mean (SD)	371.2 (325.6)	341.6 (325.6)	407.1 (353.6)	388.5 (324.2)	361.5 (330.5)	341.4 (317.4)	365.1 (351.5)	383.7 (353.1)	379.8 (339.5)	390.9 (344.7)
Ownership, public	40,571 (10.1)	8382 (12.0)	7505 (11.7)	1288 (10.4)	10,764 (11.6)	18,769 (10.9)	38,361 (10.9)	1723 (9.5)	3554 (9.9)	7798 (10.7)
Teaching hospital	59,902 (14.8)	9341 (13.3)	13,290 (20.6)	2039 (16.4)	13,906 (15.0)	22,167 (12.9)	51,735 (14.7)	3066 (16.8)	5416 (15.0)	13,671 (18.8)

Results presented as N (%) unless otherwise noted.

SD, standard deviation.

**Table 2:** Demographics, 30-Day Outcomes, Access to Health-Care Resources, and Characteristics of Admitting Health-Care Facilities Stratified by Admission Groups Within the Readmission Cohort

Variable	Gastrointestinal hemorrhage (N = 406,310)	Disorders of pancreas except malignancy (N = 69,479)	Liver disease (N = 62,557)	Esophageal disorders (N = 12,650)	Appendicitis and peritoneal infections (N = 92,673)	Gastrointestinal obstruction (N = 169,509)	Gastroenteritis and esophagitis (N = 346,333)	Inflammatory bowel disease (N = 18,226)	Peptic ulcer disease (N = 36,468)	Biliary tract disorders (N = 74,901)
Main outcome										
Observed readmission	68,204 (16.8)	10,002 (14.4)	16,359 (26.2)	2453 (19.4)	19,473 (21.0)	22,664 (13.4)	52,484 (15.2)	3022 (16.6)	6043 (16.6)	13,310 (17.8)
Demographics										
Age (y), mean (SD)	79.8 (8.2)	76.4 (8.0)	74.1 (7.0)	77.3 (8.1)	78.4 (8.3)	78.4 (8.3)	78.2 (8.3)	76.1 (7.5)	78.1 (8.2)	79.4 (8.6)
Legal sex, female	219,518 (54.0)	38,129 (54.9)	30,061 (48.1)	6506 (51.4)	58,959 (63.6)	98,309 (58.0)	234,493 (67.7)	11,565 (63.5)	20,056 (55.0)	41,353 (55.2)
Race/ethnicity										
Asian	6619 (1.6)	1174 (1.7)	1089 (1.7)	234 (1.8)	1028 (1.1)	2438 (1.4)	4625 (1.3)	172 (0.9)	631 (1.7)	1717 (2.3)
Black	45,599 (11.2)	6200 (8.9)	4106 (6.6)	1335 (10.6)	6102 (6.6)	14,389 (8.5)	29,589 (8.5)	1062 (5.8)	3047 (8.4)	5256 (7.0)
Hispanic	6054 (1.5)	1347 (1.9)	1959 (3.1)	248 (2.0)	1352 (1.5)	2623 (1.5)	6221 (1.8)	191 (1.0)	632 (1.7)	1651 (2.2)
Other/Unknown	10,764 (2.6)	2180 (3.1)	2629 (4.2)	430 (3.4)	2292 (2.5)	4746 (2.8)	8310 (2.4)	493 (2.7)	975 (2.7)	2428 (3.2)
White	337,274 (83.0)	58,578 (84.3)	52,774 (84.4)	10,403 (82.2)	81,899 (88.4)	145,313 (85.7)	297,588 (85.9)	16,308 (89.5)	31,183 (85.5)	63,849 (85.2)
Dual Medicare/Medicaid eligible	87,721 (21.6)	13,576 (19.5)	15,562 (24.9)	2903 (22.9)	18,958 (20.5)	32,525 (19.2)	73,556 (21.2)	2906 (15.9)	8614 (23.6)	16,149 (21.6)
Medical history										
Elixhauser readmission index, mean (SD)	51.6 (27.6)	41.4 (26.9)	68.9 (28.0)	55.6 (28.0)	54.5 (28.7)	41.6 (27.4)	44.2 (27.4)	41.3 (25.6)	50.9 (27.7)	46.2 (27.9)
Regional information										
Rural area	83,162 (20.5)	16,290 (23.4)	13,098 (20.9)	2540 (20.1)	20,029 (21.6)	37,029 (21.8)	70,703 (20.4)	3290 (18.1)	6854 (18.8)	15,884 (21.2)
Primary care providers per 100,000 persons, mean (SD)	73.9 (32.6)	71.9 (32.3)	72.4 (32.4)	74.1 (32.3)	73.2 (32.5)	73.7 (32.4)	73.8 (32.6)	75.2 (32.3)	74.2 (32.0)	74.6 (32.9)
Total specialists per 100,000 persons, mean (SD)	217.4 (181.7)	203.2 (174.0)	209.5 (177.7)	219.0 (178.5)	211.9 (179.5)	212.5 (178.0)	217.3 (181.2)	223.2 (181.4)	217.1 (175.0)	221.1 (187.7)
Hospital beds per 10,000 persons, mean (SD)	28.8 (22.6)	28.3 (23.4)	28.2 (22.3)	28.6 (21.7)	28.5 (21.6)	28.5 (22.8)	29.0 (23.1)	28.3 (21.9)	28.5 (21.4)	28.3 (22.0)
Distance to the closest hospital, miles, mean (SD)	4.2 (5.3)	4.3 (5.4)	4.3 (5.5)	4.3 (5.3)	4.2 (5.2)	4.1 (5.2)	4.1 (5.1)	4.2 (5.1)	4.2 (5.5)	4.2 (5.4)
Hospital information										
Number of beds, mean (SD)	383.3 (328.4)	361.2 (333.4)	429.7 (361.9)	405.7 (329.0)	369.6 (334.1)	354.6 (322.7)	371.7 (353.4)	392.2 (357.0)	393.4 (342.7)	435.1 (356.0)
Ownership, public	40,215 (9.9)	8193 (11.8)	7251 (11.6)	1316 (10.4)	10,703 (11.5)	18,125 (10.7)	37,681 (10.9)	1697 (9.3)	3579 (9.8)	7784 (10.4)
Teaching hospital	65,285 (16.1)	10,777 (15.5)	14,665 (23.4)	2354 (18.6)	14,706 (15.9)	23,998 (14.2)	53,427 (15.4)	3221 (17.7)	6142 (16.8)	17,960 (24.0)

Results presented as N (%) unless otherwise noted.

SD, standard deviation.

**Table 3:** Observed Outcomes (30-Day Mortality and Readmission) by Study Group and Neighborhood SES

Study group/outcome	High neighborhood SES [ADI 1–15]	Middle neighborhood SES [ADI 16–85]	Low neighborhood SES [ADI 86–100]	*P*
Gastrointestinal hemorrhage				
Mortality	3785/54,175 (7.0)	22,332/300,041 (7.4)	3879/49,268 (7.9)	<.001
Readmission	8300/52,585 (15.8)	50,684/302,564 (16.8)	9220/51,161 (18.0)	<.001
Disorders of pancreas except malignancy				
Mortality	318/8575 (3.7)	2387/52,672 (4.5)	421/8882 (4.7)	.001
Readmission	1133/8306 (13.6)	7516/52,269 (14.4)	1353/8904 (15.2)	.01
Liver disease				
Mortality	1570/7776 (20.2)	10,454/48,366 (21.6)	1850/8231 (22.5)	.002
Readmission	1822/7267 (25.1)	12,351/47,185 (26.2)	2186/8105 (27.0)	.03
Esophageal disorders				
Mortality	147/1647 (8.9)	846/9370 (9.0)	145/1418 (10.2)	.33
Readmission	311/1595 (19.5)	1827/9553 (19.1)	315/1502 (21.0)	.24
Appendicitis and peritoneal infections				
Mortality	768/11,761 (6.5)	5225/70,746 (7.4)	821/10,487 (7.8)	<.001
Readmission	2397/11,547 (20.8)	14,724/70,516 (20.9)	2352/10,610 (22.2)	.008
Gastrointestinal obstruction				
Mortality	1344/23,866 (5.6)	8412/128,746 (6.5)	1417/19,283 (7.3)	<.001
Readmission	2911/23,104 (12.6)	16,916/127,117 (13.3)	2837/19,288 (14.7)	<.001
Gastroenteritis and esophagitis				
Mortality	1683/45,958 (3.7)	10,552/262,229 (4.0)	1672/42,848 (3.9)	<.001
Readmission	6569/44,435 (14.8)	39,068/259,098 (15.1)	6847/42,800 (16.0)	<.001
Inflammatory bowel disease				
Mortality	90/2629 (3.4)	485/13,873 (3.5)	71/1727 (4.1)	.40
Readmission	407/2574 (15.8)	2316/13,891 (16.7)	299/1761 (17.0)	.50
Peptic ulcer disease				
Mortality	380/4937 (7.7)	2048/26,884 (7.6)	319/4238 (7.5)	.95
Readmission	746/4832 (15.4)	4486/27,231 (16.5)	811/4405 (18.4)	<.001
Biliary tract disorders				
Mortality	681/11,249 (6.1)	3695/53,582 (6.9)	622/8019 (7.8)	<.001
Readmission	2064/11,000 (18.8)	9697/55,334 (17.5)	1549/8567 (18.1)	.006

Presented as: # with outcome/# in denominator (%); in ADI, 100 indicates the most deprived neighborhood and 1 indicates the least deprived neighborhood.

Table 4 shows unadjusted and sequentially adjusted estimates of the association between neighborhood deprivation and 30-day mortality, which is stratified by admission DRG. Before adjustment, there was a moderate dose-dependent relationship between neighborhood SES and 30-day mortality for the pancreatic disorders (ADI 100 vs 1: OR 1.47, 95% CI 1.20–1.80) and gastrointestinal obstruction groups (ADI 100 vs 1: OR 1.36, 95% CI 1.21–1.52). A moderate relationship was also observed for patients from the lowest SES neighborhoods in the biliary tract disorders group (ADI 100 vs 1: OR 1.36, 95% CI 1.16–1.60). Adjusting for patient characteristics elucidated a dose-dependent relationship between area deprivation and mortality for the gastrointestinal hemorrhage, pancreatic disorders, liver disease, appendicitis and peritoneal infections, gastrointestinal obstruction, gastroenteritis and esophagitis, and biliary tract disorders groups. These relationships persisted after adjusting for access to health care for all groups except gastroenteritis and esophagitis. Subsequent adjustment for characteristics of treating hospitals had no further effect on the relationship between neighborhood deprivation and 30-day mortality. In fully adjusted models, the strongest associations between maximal neighborhood deprivation and 30-day mortality were for nonmalignant pancreatic disorders (OR 1.59, 95% CI 1.25–2.01), esophageal disorders (OR 1.50, 95% 1.02–2.21), gastrointestinal hemorrhage (OR 1.40, 95% CI 1.29–1.52), and biliary tract disorders (OR 1.40, 95% CI 1.16–1.69). Estimates from regression models for mortality where ADI was grouped info low, middle, and high neighborhood SES (Table A3) were similar to those reported above.

**Table 4:** Regression-Estimated Effects of Neighborhood SES on Odds of 30-Day Mortality

Group	ADI percentile	Model 1 (unadjusted) OR (95% CI)	Model 2 (+Patient characteristics) OR (95% CI)	Model 3 (+Health-care access) OR (95% CI)	Model 4 (+Hospital characteristics) OR (95% CI)
Gastrointestinal hemorrhage	1 (least deprived)	1.00 (Ref)	1.00 (Ref)	1.00 (Ref)	1.00 (Ref)
	15	0.99 (0.96–1.03)	1.08 (1.04–1.11)	1.07 (1.03–1.11)	1.08 (1.04–1.12)
	50	1.05 (0.99–1.11)	1.25 (1.18–1.33)	1.22 (1.15–1.30)	1.26 (1.18–1.34)
	85	1.11 (1.06–1.17)	1.37 (1.30–1.45)	1.32 (1.24–1.40)	1.36 (1.28–1.45)
	100 (most deprived)	1.11 (1.04–1.19)	1.41 (1.31–1.52)	1.35 (1.25–1.46)	1.40 (1.29–1.52)
Disorders of pancreas except malignancy	1	1.00 (Ref)	1.00 (Ref)	1.00 (Ref)	1.00 (Ref)
	15	1.15 (1.04–1.27)	1.21 (1.09–1.34)	1.18 (1.06–1.31)	1.19 (1.07–1.33)
	50	1.33 (1.12–1.57)	1.51 (1.26–1.81)	1.40 (1.17–1.69)	1.44 (1.19–1.73)
	85	1.39 (1.19–1.62)	1.63 (1.37–1.92)	1.46 (1.22–1.75)	1.49 (1.24–1.79)
	100	1.47 (1.20–1.80)	1.75 (1.40–2.19)	1.56 (1.23–1.96)	1.59 (1.25–2.01)
Liver disease	1	1.00 (Ref)	1.00 (Ref)	1.00 (Ref)	1.00 (Ref)
	15	1.04 (0.98–1.09)	1.10 (1.04–1.16)	1.09 (1.03–1.15)	1.09 (1.03–1.15)
	50	1.09 (1.00–1.20)	1.26 (1.15–1.39)	1.23 (1.11–1.36)	1.23 (1.11–1.36)
	85	1.11 (1.02–1.21)	1.33 (1.22–1.46)	1.28 (1.16–1.41)	1.27 (1.15–1.40)
	100	1.12 (1.00–1.25)	1.38 (1.22–1.55)	1.31 (1.16–1.49)	1.30 (1.14–1.47)
Esophageal disorders	1	1.00 (Ref)	1.00 (Ref)	1.00 (Ref)	1.00 (Ref)
	15	0.99 (0.85–1.14)	1.03 (0.88–1.21)	1.03 (0.87–1.21)	1.03 (0.87–1.21)
	50	0.96 (0.75–1.22)	1.10 (0.84–1.43)	1.06 (0.80–1.41)	1.07 (0.80–1.42)
	85	1.10 (0.87–1.38)	1.33 (1.03–1.72)	1.28 (0.96–1.71)	1.29 (0.96–1.72)
	100	1.24 (0.90–1.72)	1.54 (1.07–2.21)	1.49 (1.01–2.19)	1.50 (1.02–2.21)
Appendicitis and peritoneal infections	1	1.00 (Ref)	1.00 (Ref)	1.00 (Ref)	1.00 (Ref)
	15	1.04 (0.97–1.11)	1.12 (1.05–1.20)	1.10 (1.03–1.19)	1.11 (1.03–1.19)
	50	1.12 (1.00–1.25)	1.34 (1.19–1.51)	1.26 (1.12–1.42)	1.26 (1.12–1.43)
	85	1.16 (1.05–1.29)	1.45 (1.30–1.62)	1.31 (1.16–1.48)	1.31 (1.16–1.48)
	100	1.18 (1.02–1.37)	1.51 (1.29–1.77)	1.35 (1.14–1.59)	1.35 (1.14–1.59)
Gastrointestinal obstruction	1	1.00 (Ref)	1.00 (Ref)	1.00 (Ref)	1.00 (Ref)
	15	1.05 (0.99–1.11)	1.11 (1.05–1.17)	1.09 (1.03–1.15)	1.09 (1.03–1.16)
	50	1.20 (1.09–1.31)	1.40 (1.27–1.54)	1.28 (1.16–1.42)	1.30 (1.18–1.44)
	85	1.32 (1.22–1.44)	1.53 (1.40–1.67)	1.33 (1.21–1.46)	1.35 (1.22–1.48)
	100	1.36 (1.21–1.52)	1.53 (1.35–1.73)	1.31 (1.15–1.49)	1.32 (1.16–1.51)
Gastroenteritis and esophagitis	1	1.00 (Ref)	1.00 (Ref)	1.00 (Ref)	1.00 (Ref)
	15	1.00 (0.96–1.05)	1.08 (1.03–1.13)	1.07 (1.02–1.13)	1.09 (1.04–1.15)
	50	1.08 (1.00–1.16)	1.28 (1.19–1.39)	1.24 (1.14–1.34)	1.28 (1.17–1.41)
	85	1.06 (0.99–1.14)	1.33 (1.23–1.44)	1.24 (1.14–1.34)	1.28 (1.17–1.40)
	100	1.00 (0.90–1.10)	1.30 (1.17–1.44)	1.19 (1.07–1.33)	1.23 (1.09–1.38)
Inflammatory bowel disease	1	1.00 (Ref)	1.00 (Ref)	1.00 (Ref)	1.00 (Ref)
	15	0.98 (0.80–1.21)	1.08 (0.87–1.33)	1.03 (0.82–1.28)	1.01 (0.81–1.26)
	50	1.04 (0.75–1.44)	1.27 (0.89–1.80)	1.11 (0.76–1.62)	1.08 (0.74–1.58)
	85	1.25 (0.93–1.69)	1.46 (1.06–2.03)	1.23 (0.85–1.78)	1.20 (0.83–1.73)
	100	1.36 (0.88–2.10)	1.56 (0.97–2.50)	1.28 (0.77–2.14)	1.24 (0.74–2.07)
Peptic ulcer disease	1	1.00 (Ref)	1.00 (Ref)	1.00 (Ref)	1.00 (Ref)
	15	1.00 (0.90–1.10)	1.12 (1.00–1.25)	1.12 (1.00–1.25)	1.11 (0.99–1.24)
	50	0.96 (0.81–1.14)	1.28 (1.07–1.54)	1.26 (1.04–1.52)	1.24 (1.02–1.50)
	85	0.95 (0.81–1.12)	1.32 (1.11–1.57)	1.26 (1.04–1.52)	1.24 (1.02–1.50)
	100	0.96 (0.77–1.20)	1.36 (1.07–1.74)	1.29 (1.00–1.66)	1.27 (0.98–1.64)
Biliary tract disorders	1	1.00 (Ref)	1.00 (Ref)	1.00 (Ref)	1.00 (Ref)
	15	1.07 (0.99–1.15)	1.13 (1.05–1.22)	1.10 (1.01–1.19)	1.09 (1.01–1.18)
	50	1.20 (1.06–1.35)	1.44 (1.27–1.64)	1.30 (1.14–1.49)	1.29 (1.13–1.48)
	85	1.30 (1.16–1.46)	1.63 (1.44–1.84)	1.39 (1.22–1.59)	1.38 (1.21–1.58)
	100	1.36 (1.16–1.60)	1.68 (1.41–2.00)	1.41 (1.18–1.70)	1.40 (1.16–1.69)

Model 1 covariate included ADI restricted cubic spline terms only. Model 2 added covariates for age, sex, race/ethnicity, year of admission, end-stage renal disease status, and comorbid conditions. Model 3 added covariates for residence in a rural area, number of primary care providers per 100,000 persons, total number of specialists per 100,000 persons, hospital beds per 10,000 persons, and distance to the nearest hospital in miles. Model 4 added covariates for number of beds of admitting hospital, teaching status of admitting hospital, and public vs private ownership of admitting hospital.

Table 5 shows unadjusted and sequentially adjusted estimates of the effect of neighborhood deprivation on 30-day readmission, stratified by admission group. Before adjustment, there was a moderate dose-dependent relationship between neighborhood SES and 30-day readmission for the gastrointestinal hemorrhage group. An association was also observed for those from the lowest neighborhood SES for patients in the gastrointestinal obstruction, gastroenteritis and esophagitis, and peptic ulcer disease groups. These associations were lost following subsequent adjustments for patient characteristics, metrics of access to health care, and characteristics of admitting hospitals. Estimates from regression models for readmission where ADI was grouped info low, middle, and high neighborhood SES (Table A4) were similar to those reported above.

**Table 5:** Regression-Estimated Effects of Neighborhood SES on Odds of 30-Day Readmission

Group	ADI percentile	Model 1 (unadjusted) OR (95% CI)	Model 2 (+Patient characteristics) OR (95% CI)	Model 3 (+Healthcare access) OR (95% CI)	Model 4 (+Hospital characteristics) OR (95% CI)
Gastrointestinal hemorrhage	1	1.00 (Ref)	1.00 (Ref)	1.00 (Ref)	1.00 (Ref)
	15	1.05 (1.02–1.07)	1.02 (1.00–1.05)	1.03 (1.00–1.05)	1.03 (1.01–1.06)
	50	1.11 (1.07–1.16)	1.05 (1.01–1.09)	1.06 (1.02–1.10)	1.07 (1.03–1.12)
	85	1.19 (1.14–1.24)	1.07 (1.03–1.11)	1.07 (1.03–1.12)	1.08 (1.04–1.13)
	100	1.26 (1.20–1.33)	1.08 (1.03–1.14)	1.09 (1.03–1.15)	1.10 (1.04–1.16)
Disorders of pancreas except malignancy	1	1.00 (Ref)	1.00 (Ref)	1.00 (Ref)	1.00 (Ref)
	15	1.08 (1.02–1.15)	1.08 (1.01–1.14)	1.09 (1.02–1.15)	1.09 (1.03–1.16)
	50	1.16 (1.05–1.29)	1.15 (1.04–1.27)	1.17 (1.05–1.30)	1.18 (1.06–1.32)
	85	1.18 (1.08–1.30)	1.11 (1.01–1.22)	1.13 (1.01–1.25)	1.14 (1.02–1.26)
	100	1.24 (1.09–1.41)	1.10 (0.97–1.26)	1.12 (0.97–1.29)	1.13 (0.98–1.30)
Liver disease	1	1.00 (Ref)	1.00 (Ref)	1.00 (Ref)	1.00 (Ref)
	15	1.04 (1.00–1.10)	1.02 (0.97–1.07)	1.03 (0.98–1.08)	1.03 (0.98–1.09)
	50	1.08 (1.00–1.17)	1.02 (0.94–1.11)	1.06 (0.97–1.15)	1.07 (0.98–1.16)
	85	1.10 (1.02–1.18)	1.00 (0.93–1.08)	1.06 (0.98–1.15)	1.07 (0.98–1.16)
	100	1.13 (1.02–1.26)	1.01 (0.91–1.12)	1.07 (0.96–1.20)	1.08 (0.97–1.21)
Esophageal disorders	1	1.00 (Ref)	1.00 (Ref)	1.00 (Ref)	1.00 (Ref)
	15	0.96 (0.85–1.08)	0.93 (0.82–1.06)	0.94 (0.83–1.07)	0.95 (0.84–1.09)
	50	0.95 (0.78–1.17)	0.90 (0.72–1.11)	0.95 (0.76–1.18)	0.96 (0.78–1.20)
	85	1.05 (0.87–1.26)	0.95 (0.78–1.16)	1.03 (0.84–1.27)	1.05 (0.85–1.29)
	100	1.08 (0.83–1.40)	0.96 (0.73–1.25)	1.04 (0.79–1.37)	1.06 (0.80–1.40)
Appendicitis and peritoneal infections	1	1.00 (Ref)	1.00 (Ref)	1.00 (Ref)	1.00 (Ref)
	15	1.00 (0.96–1.05)	0.99 (0.95–1.04)	0.99 (0.95–1.04)	1.00 (0.95–1.04)
	50	1.03 (0.96–1.11)	1.01 (0.94–1.09)	1.02 (0.94–1.10)	1.02 (0.94–1.11)
	85	1.10 (1.02–1.18)	1.03 (0.96–1.10)	1.03 (0.95–1.11)	1.04 (0.96–1.12)
	100	1.13 (1.03–1.24)	1.01 (0.92–1.12)	1.02 (0.92–1.13)	1.02 (0.92–1.14)
Gastrointestinal obstruction	1	1.00 (Ref)	1.00 (Ref)	1.00 (Ref)	1.00 (Ref)
	15	1.02 (0.98–1.06)	1.00 (0.97–1.04)	1.00 (0.96–1.04)	1.01 (0.97–1.05)
	50	1.06 (1.00–1.13)	1.03 (0.97–1.10)	1.02 (0.95–1.08)	1.03 (0.96–1.10)
	85	1.16 (1.10–1.23)	1.07 (1.01–1.13)	1.04 (0.97–1.11)	1.05 (0.98–1.12)
	100	1.24 (1.14–1.34)	1.08 (0.99–1.17)	1.04 (0.95–1.14)	1.06 (0.97–1.16)
Gastroenteritis and esophagitis	1	1.00 (Ref)	1.00 (Ref)	1.00 (Ref)	1.00 (Ref)
	15	1.03 (1.00–1.05)	1.01 (0.99–1.04)	1.01 (0.99–1.04)	1.02 (0.99–1.05)
	50	1.05 (1.00–1.09)	1.01 (0.97–1.06)	1.02 (0.98–1.07)	1.03 (0.99–1.08)
	85	1.11 (1.06–1.15)	1.02 (0.98–1.06)	1.03 (0.98–1.07)	1.04 (0.99–1.08)
	100	1.17 (1.11–1.23)	1.03 (0.98–1.09)	1.04 (0.98–1.10)	1.05 (0.99–1.11)
Inflammatory bowel disease	1	1.00 (Ref)	1.00 (Ref)	1.00 (Ref)	1.00 (Ref)
	15	0.99 (0.90–1.09)	0.99 (0.89–1.09)	0.97 (0.87–1.07)	0.97 (0.87–1.07)
	50	1.07 (0.91–1.26)	1.07 (0.90–1.26)	1.01 (0.84–1.20)	1.01 (0.84–1.20)
	85	1.13 (0.97–1.32)	1.07 (0.91–1.26)	1.00 (0.83–1.19)	1.00 (0.83–1.19)
	100	1.09 (0.88–1.36)	1.00 (0.79–1.25)	0.92 (0.73–1.18)	0.92 (0.72–1.17)
Peptic ulcer disease	1	1.00 (Ref)	1.00 (Ref)	1.00 (Ref)	1.00 (Ref)
	15	1.07 (1.00–1.15)	1.07 (0.99–1.15)	1.06 (0.98–1.14)	1.07 (0.99–1.15)
	50	1.15 (1.02–1.30)	1.15 (1.01–1.30)	1.14 (1.00–1.29)	1.16 (1.02–1.32)
	85	1.29 (1.15–1.44)	1.22 (1.08–1.37)	1.22 (1.07–1.38)	1.24 (1.09–1.41)
	100	1.44 (1.24–1.67)	1.29 (1.10–1.51)	1.29 (1.09–1.53)	1.32 (1.11–1.56)
Biliary tract disorders	1	1.00 (Ref)	1.00 (Ref)	1.00 (Ref)	1.00 (Ref)
	15	0.96 (0.92–1.01)	0.95 (0.91–1.00)	0.95 (0.91–1.00)	0.95 (0.91–1.00)
	50	0.91 (0.84–0.98)	0.91 (0.84–0.98)	0.90 (0.83–0.98)	0.90 (0.83–0.98)
	85	0.94 (0.87–1.01)	0.91 (0.85–0.98)	0.90 (0.83–0.98)	0.91 (0.83–0.98)
	100	0.97 (0.87–1.07)	0.91 (0.82–1.02)	0.90 (0.81–1.01)	0.91 (0.81–1.01)

Model 1 covariate included ADI restricted cubic spline terms only. Model 2 added covariates for age, sex, race/ethnicity, year of admission, end-stage renal disease status, and comorbid conditions. Model 3 added covariates for residence in a rural area, number of primary care providers per 100,000 persons, total number of specialists per 100,000 persons, hospital beds per 10,000 persons, and distance to the nearest hospital in miles. Model 4 added covariates for number of beds of admitting hospital, teaching status of admitting hospital, and public vs private ownership of admitting hospital.

**Figure 1:**
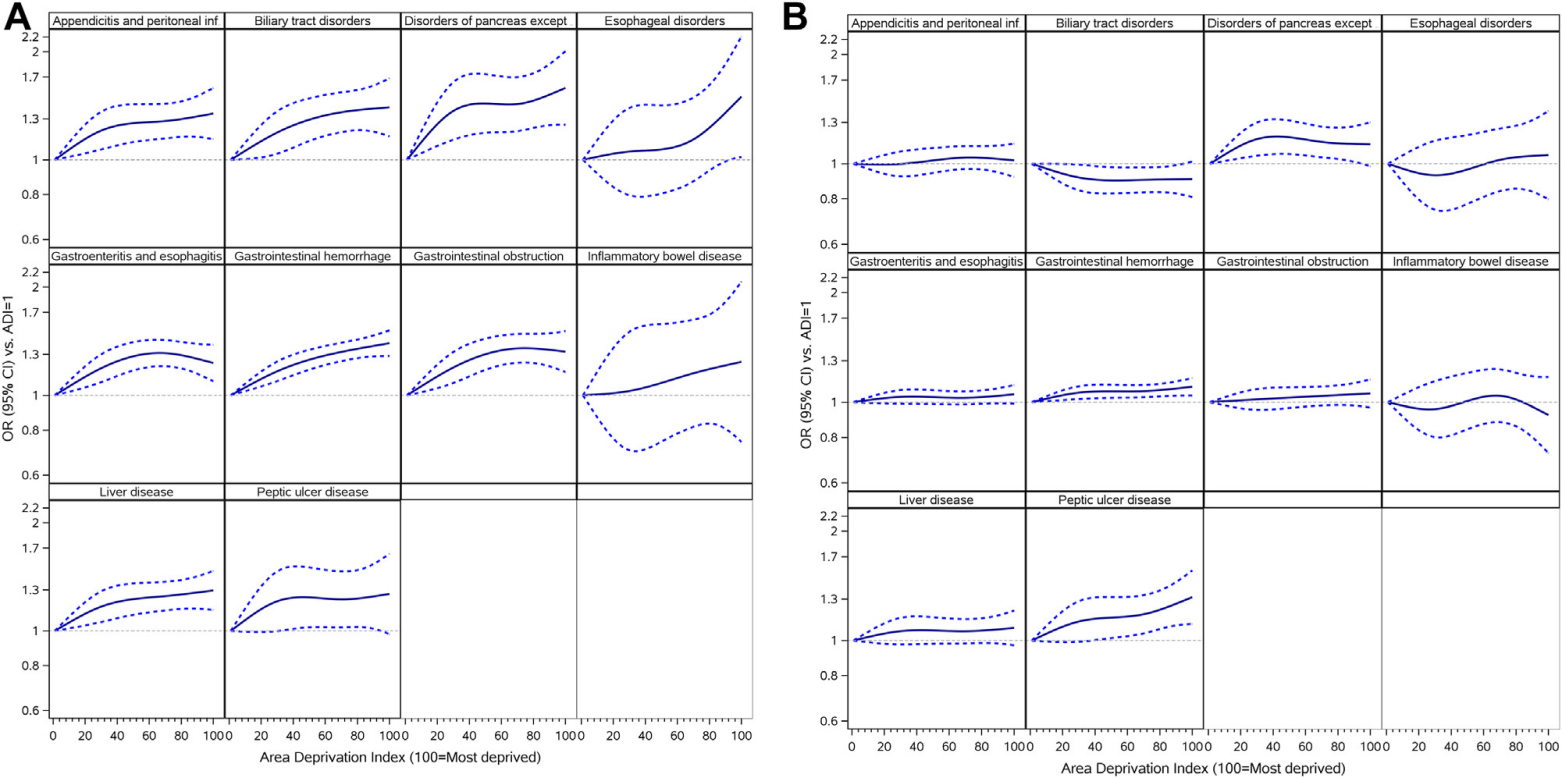
Adjusted∗ associations between neighborhood-level SES and 30-day mortality (left) and readmission (right) for patients admitted with gastrointestinal disease. ∗Adjustment covariates included age, sex, Medicare–Medicaid dual-eligibility status, end-stage renal disease status, discharge year, Elixhauser comorbidity conditions, residence in a rural area, number of primary care providers per 100,000 persons, total number of specialists per 100,000 persons, hospital beds per 10,000 persons, distance to the nearest hospital in miles, number of beds of admitting hospital, teaching status of admitting hospital, and public vs private ownership of admitting hospital.

Fully adjusted associations between neighborhood deprivation and 30-day mortality and readmission for all groups are depicted graphically in Figure 1. Within the mortality cohort, the association is broadly linear for all groups and strongest between the least deprived and moderately deprived (ADI 0–50) neighborhoods for all diagnosis groups except esophageal disorders and inflammatory bowel disease. For the gastrointestinal obstruction and gastroenteritis and esophagitis groups, this relationship slightly inverts between the moderately deprived and most deprived (ADI 50–100) neighborhoods. In contrast, for the readmission cohort, the model shows no association after full adjustment.

## Discussion

We sought to characterize the relationships between neighborhood SES and 30-day mortality and readmission for patients with common gastrointestinal diseases in a large nationwide cohort of Medicare beneficiaries. Our study demonstrated an independent association between neighborhood deprivation and 30-day mortality for patients with common gastrointestinal diseases. These relationships persisted and were not significantly attenuated after adjusting for individual poverty, demographics and comorbidities, access to health-care resources, and characteristics of treating facilities. In contrast, we did not observe an independent association between neighborhood deprivation and 30-day readmission for patients before or after sequential adjustment. Our findings highlight the need for a focus on and funding for further investigation and better understanding of the underlying mechanisms of and contributors to gastrointestinal health disparities, a core element of the American Gastroenterological Association advocacy agenda.^20^

There is limited prior research evaluating the relationships between neighborhood deprivation and outcomes for patients with noncancerous gastrointestinal diseases. Drawing from multistate survey data, one study describes a strong association between area-level deprivation and mortality from chronic liver disease.^21^ This association persisted after adjusting for age, race, individual health factors, and other health behaviors; however, this model did not account for individual poverty or access to health-care resources. Research has also shown disproportionately high rates of hepatitis C-related mortality in areas with multiple indicators of geographic disparity; however, this prior study was not conducted on the level of individual patients.^22^ Another study described an association between risk of progression to perforated appendicitis and neighborhood social vulnerability among older patients.^23^ Similarly, lower incidence of acute appendicitis has been associated with markers of high area SES, as indicated by prevalence of college education, median income, and per capita income.^24^ However, these studies have been restricted to select states, cities, or individual academic medical centers and have focused on individual disease processes. Our observed association between area-level deprivation and mortality from liver disease and appendicitis-related disease in a national sample extends and generalizes the findings of this prior work and confirms that much of this effect is likely independent of individual poverty, demographics (eg, race/ethnicity), medical comorbidities, access to health-care resources, and measured characteristics of treating health-care facilities.

A range of interrelated neighborhood, environmental, and behavioral factors may drive disparities in gastrointestinal disease more broadly. Alcohol consumption is one particularly important contributor to several groups of gastrointestinal disease addressed in our study, including liver disease, pancreatitis and biliary disease, peptic ulcer disease, and gastrointestinal hemorrhage. The relationships between neighborhood SES, alcohol availability, alcohol consumption, and negative consequences of drinking are complex and mediated by social, cultural, and environmental factors. While people of low individual SES appear to bear the burden of health consequences related to alcohol consumption,^25^ individuals from the least deprived neighborhoods are more likely to consume alcohol heavily, even though it is the most deprived neighborhoods which have higher alcohol outlet density.^26^ Additionally, alcohol outlet density is not associated with current use of alcohol but rather with a higher quantity of weekly alcohol consumption among men who already consume heavily.^27^ It is also important to account for broader socioeconomic context: it has been suggested that the effect of neighborhood SES may be moderated by prodrinking attitudes, availability, and patterns of drinking, as well as the burden of stress and coping present in low-SES neighborhoods, including that from structural and individual racism.^28^^,^^29^ Further work is warranted to better understand the mechanisms through which alcohol consumption negatively impacts health for individuals from disadvantaged neighborhoods and the added burden placed on marginalized individuals in these contexts.

Nutritional disparities related to individual and neighborhood SES may also be especially relevant for diseases of the gastrointestinal system (eg, in trajectories following admission for pancreatic or liver disease). Food insecurity, a public health issue which has reached particular crisis levels during the COVID-19 pandemic, is closely related to neighborhood SES and may contribute to the inequities observed in our study.^30^ Recent data has shown that food insecurity is associated with mortality in adults with nonalcoholic fatty liver disease and fibrosis.^31^ The complex interplay of food insecurity, comorbid cardiometabolic disease, and gastrointestinal disease warrants further investigation, including in the posthospitalization period.

Delayed presentation for care, whether driven by suboptimal transportation access, health-care avoidance (eg, as a result of historical discrimination), or other socioeconomically informed factors may also play a role in disparate outcomes for gastrointestinal disease according to area deprivation. Our study included a variety of diseases which can present acutely or chronically, but for which timeliness of interventions is often pivotal. Future investigation may elucidate the degree to which delayed presentation may mediate these relationships.

Further, we found an association between neighborhood deprivation and 30-day mortality for patients admitted with noncancerous pancreatic disease, biliary tract disorders, and gastroenteritis (including diverticulitis). Our findings may be related to disparities in the surgical management of certain gastrointestinal conditions. Lower individual SES has previously been associated with lower likelihood of same-admission cholecystectomy following endoscopic retrograde cholangiopancreatography for patients with gallstone pancreatitis.^32^ Moreover, low SES patients admitted with diverticular disease are more likely to present emergently and have worse disease, while being less likely to receive surgery, compared to high SES counterparts.^33^ At the area level, patients from low-SES neighborhoods with ostomies placed for diverticulitis were less likely to have this temporary measure reversed and more likely to die, a relationship particularly pronounced for non-Hispanic Black patients.^34^

There are several reasons why we may have observed an association between neighborhood deprivation and 30-day mortality and not 30-day readmission for many conditions. We only observed adjusted associations between neighborhood SES and 30-day readmission for patients admitted with gastrointestinal hemorrhage or peptic ulcer disease, both of which are acute medical conditions for which short-term readmission for recurrence is common. By contrast, other disorders (such as inflammatory bowel disease) reflect acute manifestations of more chronic processes or are acute events for which curative therapy is often employed (appendicitis). It is possible that neighborhood deprivation might exhibit an association with readmissions over longer durations for these conditions. Similarly, the lack of association between neighborhood deprivation and mortality for inflammatory bowel disease could be due to similar issues related to chronicity of disease burden.

The increased risks of mortality associated with neighborhood deprivation may be comparable to evidence-based, guideline-recommended interventions in gastroenterology. For example, the use of high-dose proton pump inhibitor therapy after successful endoscopic treatment of bleeding gastrointestinal ulcer represents a staple of management (receiving the strongest recommendation and highest level of evidence in the 2021 ACG Upper Gastrointestinal and Ulcer Bleeding guideline).^35^ In this patient population, the relative risk reduction of mortality associated with receipt of proton pump inhibitors therapy vs placebo has been estimated at 57%, compared with the 40% relative risk increase associated with residence in a highly deprived neighborhood.^35^

Limitations to our study include the use of medical claims data, which may not capture the entirety of a patient’s baseline medical risk, and restriction to the Medicare population, which may not be fully generalizable to a younger, uninsured, or otherwise insured population. Our analyses use DRGs, which limit the ability to detail more nuanced associations of specific diseases with 30-day mortality and readmission. Our study also used Medicare–Medicaid dual-eligibility as a dichotomous measure of individual SES, a coarse metric. Further, the ADI, while widely used, certainly does not capture the entirety of neighborhood-level exposures which may influence gastrointestinal disease outcomes. Specifically, it does not explicitly include important social and environmental characteristics such as racial segregation, crime, or green space; furthermore, the ADI places a heavy reliance on housing value, which could mean it insufficiently captures other critical dimensions of neighborhood environment.

## Conclusion

In this study, we demonstrate that neighborhood deprivation is independently associated with 30-day mortality for patients with common gastrointestinal diseases. The widely disparate mortality outcomes by neighborhood deprivation level in the Medicare population, which persist despite controls for demographics, comorbidities, access to health-care resources, and treatment facility characteristics, is a call to action for health equity. Our study highlights the need to understand the underlying mechanisms of neighborhood deprivation on worse gastrointestinal disease outcomes and to prioritize interventions that address these disparities.

## Supplementary Materials

Tables A1–5
