# Supplementary material for: Neighborhood Socioeconomic Deprivation and 30-Day Outcomes After Admission for Common Gastrointestinal Conditions: A Large Nationwide Study
Source: Gastro Hep Adv. 2025 Jan 9;4(5):100614. doi: 10.1016/j.gastha.2025.100614 (PMC12008582; doi:10.1016/j.gastha.2025.100614)
Supplement: Tables A1–5 [file mmc1.docx]

**Supplementary Table 1.** Demographics, 30-day outcomes, access to healthcare resources, and characteristics of admitting healthcare facilities, stratified by neighborhood socioeconomic status group within the **mortality** cohort.

| **Variable** | **High neighborhood SES / ADI 1-15**  **(N = 172,573)** | **Middle neighborhood SES / ADI 16-85**  **(N = 966,509)** | **Low neighborhood SES / ADI 86-100**  **(N = 154,401)** | |
| --- | --- | --- | --- | --- |
| Main Outcome |  |  | |  |
| Observed mortality | 10,766 (6.2) | 66,436 (6.9) | | 11,217 (7.3) |
| Demographics |  |  | |  |
| Age (years), Mean (SD) | 79.8 (8.5) | 78.4 (8.3) | | 77.6 (8.2) |
| Legal Sex, Female | 98,250 (56.9) | 568,317 (58.8) | | 94,290 (61.1) |
| Race/ethnicity |  |  | |  |
| Asian | 8,880 (5.1) | 10,863 (1.1) | | 652 (0.4) |
| Black | 9,014 (5.2) | 75,812 (7.8) | | 33,886 (21.9) |
| Hispanic | 3,231 (1.9) | 16,212 (1.7) | | 3,318 (2.1) |
| Other/Unknown | 8,691 (5.0) | 22,684 (2.3) | | 3,760 (2.4) |
| White | 142,757 (82.7) | 840,938 (87.0) | | 112,785 (73.0) |
| Dual Medicare/Medicaid eligible | 33,209 (19.2) | 186,938 (19.3) | | 53,613 (34.7) |
| Medical history |  |  | |  |
| Elixhauser mortality index, Mean (SD) | 19.6 (16.1) | 19.1 (15.7) | | 19.8 (15.6) |
| Regional information |  |  | |  |
| Rural area | 2,516 (1.5) | 185,411 (19.2) | | 65,284 (42.3) |
| Primary care providers per 100,000 persons, Mean (SD) | 95.5 (29.3) | 72.6 (31.5) | | 60.8 (30.9) |
| Total specialists per 100,000 persons, Mean (SD) | 343.4 (198.7) | 207.5 (171.0) | | 153.5 (156.3) |
| Hospital beds per 10,000 persons, Mean (SD) | 26.5 (13.8) | 28.4 (21.9) | | 33.7 (31.0) |
| Distance to the closest hospital, miles, Mean (SD) | 2.8 (3.0) | 4.3 (5.2) | | 4.7 (6.8) |
| Hospital information |  |  | |  |
| Number of beds, Mean (SD) | 433.9 (385.0) | 360.4 (329.4) | | 331.3 (305.0) |
| Ownership, Public | 14,143 (8.2) | 100,718 (10.4) | | 23,854 (15.4) |
| Teaching hospital | 46,167 (26.8) | 131,469 (13.6) | | 16,897 (10.9) |

Results presented as N (%) unless otherwise noted. ADI = Area Deprivation Index; SES = Socioeconomic status; SD = Standard Deviation. Differences between groups were significant for all variables at p<.001.

**Supplementary Table 2.** Demographics, 30-day outcomes, access to healthcare resources, and characteristics of admitting healthcare facilities, stratified by neighborhood socioeconomic status group within the **readmission** cohort.

| **Variable** | **High neighborhood SES / ADI 1-15**  **(N = 167,245)** | **Middle neighborhood SES / ADI 16-85**  **(N = 964,758)** | **Low neighborhood SES / ADI 86-100**  **(N = 157,103)** |
| --- | --- | --- | --- |
| Main Outcome |  |  |  |
| Observed readmission | 26,660 (15.9) | 159,585 (16.5) | 27,769 (17.7) |
| Demographics |  |  |  |
| Age (years), Mean (SD) | 79.8 (8.5) | 78.4 (8.3) | 77.6 (8.2) |
| Legal Sex, Female | 95,429 (57.1) | 567,578 (58.8) | 95,942 (61.1) |
| Race/ethnicity |  |  |  |
| Asian | 8,505 (5.1) | 10,582 (1.1) | 640 (0.4) |
| Black | 8,716 (5.2) | 74,342 (7.7) | 33,627 (21.4) |
| Hispanic | 3,126 (1.9) | 15,840 (1.6) | 3,312 (2.1) |
| Other/Unknown | 8,379 (5.0) | 22,804 (2.4) | 4,064 (2.6) |
| White | 138,519 (82.8) | 841,190 (87.2) | 115,460 (73.5) |
| Dual Medicare/Medicaid eligible | 32,005 (19.1) | 186,154 (19.3) | 54,311 (34.6) |
| Medical history |  |  |  |
| Elixhauser readmission index, Mean (SD) | 47.5 (28.5) | 48.1 (28.2) | 51.1 (28.5) |
| Regional information |  |  |  |
| Rural area | 2,585 (1.5) | 196,723 (20.4) | 69,571 (44.3) |
| Primary care providers per 100,000 persons, Mean (SD) | 95.5 (29.3) | 72.1 (31.5) | 59.9 (30.8) |
| Total specialists per 100,000 persons, Mean (SD) | 343.0 (198.5) | 204.3 (170.7) | 148.6 (154.6) |
| Hospital beds per 10,000 persons, Mean (SD) | 26.5 (13.8) | 28.3 (22.0) | 33.4 (31.6) |
| Distance to the closest hospital, miles, Mean (SD) | 2.8 (3.0) | 4.3 (5.2) | 4.8 (6.9) |
| Hospital information |  |  |  |
| Number of beds, Mean (SD) | 440.0 (388.6) | 374.2 (334.0) | 352.6 (312.9) |
| Ownership, Public | 13,701 (8.2) | 99,396 (10.3) | 23,447 (14.9) |
| Teaching hospital | 46,243 (27.6) | 146,097 (15.1) | 20,195 (12.9) |

Results presented as N (%) unless otherwise noted. ADI = Area Deprivation Index; SES = Socioeconomic status; SD = Standard Deviation. Differences between groups were significant for all variables at p<.001.

**Supplementary Table 3.** Regression-estimated effects of neighborhood socioeconomic status on odds of 30-day **mortality.**

| **Group / Neighborhood SES group** | **Model 1**  **(Unadjusted)**  **OR (95% CI)** | **Model 2**  **(+Patient characteristics)**  **OR (95% CI)** | **Model 3**  **(+Healthcare access)**  **OR (95% CI)** | **Model 4**  **(+Hospital characteristics)**  **OR (95% CI)** |
| --- | --- | --- | --- | --- |
| Gastrointestinal hemorrhage |  |  |  |  |
| High neighborhood SES / ADI 1-15 | 1.00 (Ref) | 1.00 (Ref) | 1.00 (Ref) | 1.00 (Ref) |
| Middle neighborhood SES / ADI 16-85 | 1.05 (1.02, 1.09) | 1.19 (1.14, 1.23) | 1.15 (1.11, 1.20) | 1.18 (1.13, 1.23) |
| Low neighborhood SES / ADI 86-100 | 1.11 (1.06, 1.17) | 1.32 (1.25, 1.39) | 1.25 (1.18, 1.32) | 1.29 (1.22, 1.37) |
|  |  |  |  |  |
| Disorders of pancreas except malignancy |  |  |  |  |
| High neighborhood SES / ADI 1-15 | 1.00 (Ref) | 1.00 (Ref) | 1.00 (Ref) | 1.00 (Ref) |
| Middle neighborhood SES / ADI 16-85 | 1.22 (1.08, 1.37) | 1.34 (1.18, 1.53) | 1.25 (1.10, 1.43) | 1.27 (1.11, 1.46) |
| Low neighborhood SES / ADI 86-100 | 1.27 (1.10, 1.48) | 1.44 (1.22, 1.69) | 1.28 (1.08, 1.51) | 1.29 (1.09, 1.54) |
|  |  |  |  |  |
| Liver disease |  |  |  |  |
| High neighborhood SES / ADI 1-15 | 1.00 (Ref) | 1.00 (Ref) | 1.00 (Ref) | 1.00 (Ref) |
| Middle neighborhood SES / ADI 16-85 | 1.08 (1.01, 1.15) | 1.18 (1.11, 1.26) | 1.15 (1.08, 1.23) | 1.15 (1.07, 1.23) |
| Low neighborhood SES / ADI 86-100 | 1.13 (1.05, 1.23) | 1.32 (1.22, 1.44) | 1.26 (1.16, 1.38) | 1.26 (1.15, 1.37) |
|  |  |  |  |  |
| Esophageal disorders |  |  |  |  |
| High neighborhood SES / ADI 1-15 | 1.00 (Ref) | 1.00 (Ref) | 1.00 (Ref) | 1.00 (Ref) |
| Middle neighborhood SES / ADI 16-85 | 1.01 (0.85, 1.20) | 1.12 (0.92, 1.35) | 1.08 (0.88, 1.31) | 1.08 (0.88, 1.32) |
| Low neighborhood SES / ADI 86-100 | 1.16 (0.91, 1.47) | 1.35 (1.04, 1.77) | 1.28 (0.96, 1.69) | 1.28 (0.97, 1.70) |
|  |  |  |  |  |
| Appendicitis and peritoneal infections |  |  |  |  |
| High neighborhood SES / ADI 1-15 | 1.00 (Ref) | 1.00 (Ref) | 1.00 (Ref) | 1.00 (Ref) |
| Middle neighborhood SES / ADI 16-85 | 1.13 (1.05, 1.23) | 1.30 (1.20, 1.42) | 1.23 (1.13, 1.34) | 1.23 (1.13, 1.35) |
| Low neighborhood SES / ADI 86-100 | 1.21 (1.09, 1.34) | 1.46 (1.30, 1.63) | 1.31 (1.17, 1.47) | 1.31 (1.17, 1.47) |
|  |  |  |  |  |
| Gastrointestinal obstruction |  |  |  |  |
| High neighborhood SES / ADI 1-15 | 1.00 (Ref) | 1.00 (Ref) | 1.00 (Ref) | 1.00 (Ref) |
| Middle neighborhood SES / ADI 16-85 | 1.16 (1.09, 1.24) | 1.29 (1.21, 1.38) | 1.19 (1.11, 1.28) | 1.21 (1.12, 1.30) |
| Low neighborhood SES / ADI 86-100 | 1.31 (1.21, 1.42) | 1.44 (1.32, 1.58) | 1.24 (1.13, 1.36) | 1.25 (1.14, 1.38) |
|  |  |  |  |  |
| Gastroenteritis and esophagitis |  |  |  |  |
| High neighborhood SES / ADI 1-15 | 1.00 (Ref) | 1.00 (Ref) | 1.00 (Ref) | 1.00 (Ref) |
| Middle neighborhood SES / ADI 16-85 | 1.08 (1.02, 1.14) | 1.22 (1.15, 1.29) | 1.17 (1.10, 1.24) | 1.20 (1.13, 1.29) |
| Low neighborhood SES / ADI 86-100 | 1.04 (0.97, 1.12) | 1.25 (1.16, 1.35) | 1.15 (1.06, 1.25) | 1.18 (1.09, 1.29) |
|  |  |  |  |  |
| Inflammatory bowel disease |  |  |  |  |
| High neighborhood SES / ADI 1-15 | 1.00 (Ref) | 1.00 (Ref) | 1.00 (Ref) | 1.00 (Ref) |
| Middle neighborhood SES / ADI 16-85 | 1.02 (0.82, 1.28) | 1.16 (0.91, 1.47) | 1.03 (0.80, 1.33) | 1.01 (0.78, 1.31) |
| Low neighborhood SES / ADI 86-100 | 1.21 (0.88, 1.65) | 1.34 (0.95, 1.89) | 1.11 (0.76, 1.61) | 1.08 (0.75, 1.57) |
|  |  |  |  |  |
| Peptic ulcer disease |  |  |  |  |
| High neighborhood SES / ADI 1-15 | 1.00 (Ref) | 1.00 (Ref) | 1.00 (Ref) | 1.00 (Ref) |
| Middle neighborhood SES / ADI 16-85 | 0.99 (0.87, 1.12) | 1.21 (1.06, 1.38) | 1.18 (1.03, 1.36) | 1.17 (1.02, 1.35) |
| Low neighborhood SES / ADI 86-100 | 0.98 (0.83, 1.15) | 1.30 (1.09, 1.55) | 1.23 (1.02, 1.49) | 1.22 (1.00, 1.47) |
|  |  |  |  |  |
| Biliary tract disorders |  |  |  |  |
| High neighborhood SES / ADI 1-15 | 1.00 (Ref) | 1.00 (Ref) | 1.00 (Ref) | 1.00 (Ref) |
| Middle neighborhood SES / ADI 16-85 | 1.13 (1.04, 1.22) | 1.29 (1.18, 1.41) | 1.18 (1.08, 1.29) | 1.18 (1.07, 1.30) |
| Low neighborhood SES / ADI 86-100 | 1.27 (1.14, 1.43) | 1.51 (1.34, 1.71) | 1.28 (1.12, 1.46) | 1.28 (1.12, 1.46) |

ADI = Area Deprivation Index; CI = Confidence interval; OR = Odds ratio

Model 1 covariate included ADI restricted cubic spline terms only. Model 2 added covariates for age, sex, race/ethnicity, year of admission, end-stage renal disease status, and comorbid conditions. Model 3 added covariates for residence in a rural area, number of primary care providers per 100,000 persons, total number of specialists per 100,000 persons, hospital beds per 10,000 persons, and distance to the nearest hospital in miles. Model 4 added covariates for number of beds of admitting hospital, teaching status of admitting hospital, and public vs. private ownership of admitting hospital.

**Supplementary Table 4.** Regression-estimated effects of neighborhood socioeconomic status on odds of 30-day **readmission.**

| **Group / Neighborhood SES group** | **Model 1**  **(Unadjusted)**  **OR (95% CI)** | **Model 2**  **(+Patient characteristics)**  **OR (95% CI)** | **Model 3**  **(+Healthcare access)**  **OR (95% CI)** | **Model 4**  **(+Hospital characteristics)**  **OR (95% CI)** |
| --- | --- | --- | --- | --- |
| Gastrointestinal hemorrhage |  |  |  |  |
| High neighborhood SES / ADI 1-15 | 1.00 (Ref) | 1.00 (Ref) | 1.00 (Ref) | 1.00 (Ref) |
| Middle neighborhood SES / ADI 16-85 | 1.08 (1.05, 1.11) | 1.04 (1.01, 1.07) | 1.04 (1.01, 1.07) | 1.05 (1.02, 1.08) |
| Low neighborhood SES / ADI 86-100 | 1.18 (1.14, 1.23) | 1.06 (1.02, 1.10) | 1.06 (1.02, 1.11) | 1.07 (1.03, 1.12) |
|  |  |  |  |  |
| Disorders of pancreas except malignancy |  |  |  |  |
| High neighborhood SES / ADI 1-15 | 1.00 (Ref) | 1.00 (Ref) | 1.00 (Ref) | 1.00 (Ref) |
| Middle neighborhood SES / ADI 16-85 | 1.07 (0.99, 1.15) | 1.06 (0.98, 1.14) | 1.07 (0.99, 1.15) | 1.07 (1.00, 1.16) |
| Low neighborhood SES / ADI 86-100 | 1.14 (1.04, 1.24) | 1.05 (0.96, 1.15) | 1.06 (0.96, 1.17) | 1.07 (0.97, 1.18) |
|  |  |  |  |  |
| Liver disease |  |  |  |  |
| High neighborhood SES / ADI 1-15 | 1.00 (Ref) | 1.00 (Ref) | 1.00 (Ref) | 1.00 (Ref) |
| Middle neighborhood SES / ADI 16-85 | 1.07 (1.01, 1.13) | 1.02 (0.97, 1.08) | 1.05 (0.99, 1.12) | 1.06 (1.00, 1.12) |
| Low neighborhood SES / ADI 86-100 | 1.11 (1.03, 1.19) | 1.02 (0.95, 1.10) | 1.08 (0.99, 1.17) | 1.08 (1.00, 1.17) |
|  |  |  |  |  |
| Esophageal disorders |  |  |  |  |
| High neighborhood SES / ADI 1-15 | 1.00 (Ref) | 1.00 (Ref) | 1.00 (Ref) | 1.00 (Ref) |
| Middle neighborhood SES / ADI 16-85 | 0.98 (0.85, 1.12) | 0.94 (0.81, 1.09) | 0.97 (0.83, 1.13) | 0.98 (0.84, 1.14) |
| Low neighborhood SES / ADI 86-100 | 1.09 (0.91, 1.32) | 1.00 (0.83, 1.21) | 1.06 (0.86, 1.29) | 1.07 (0.87, 1.31) |
|  |  |  |  |  |
| Appendicitis and peritoneal infections |  |  |  |  |
| High neighborhood SES / ADI 1-15 | 1.00 (Ref) | 1.00 (Ref) | 1.00 (Ref) | 1.00 (Ref) |
| Middle neighborhood SES / ADI 16-85 | 1.02 (0.96, 1.07) | 1.00 (0.95, 1.06) | 1.00 (0.94, 1.06) | 1.00 (0.95, 1.06) |
| Low neighborhood SES / ADI 86-100 | 1.10 (1.02, 1.17) | 1.01 (0.94, 1.09) | 1.01 (0.93, 1.08) | 1.01 (0.94, 1.09) |
|  |  |  |  |  |
| Gastrointestinal obstruction |  |  |  |  |
| High neighborhood SES / ADI 1-15 | 1.00 (Ref) | 1.00 (Ref) | 1.00 (Ref) | 1.00 (Ref) |
| Middle neighborhood SES / ADI 16-85 | 1.07 (1.03, 1.12) | 1.05 (1.01, 1.10) | 1.04 (0.99, 1.08) | 1.04 (1.00, 1.09) |
| Low neighborhood SES / ADI 86-100 | 1.20 (1.14, 1.27) | 1.09 (1.03, 1.16) | 1.06 (0.99, 1.13) | 1.07 (1.00, 1.14) |
|  |  |  |  |  |
| Gastroenteritis and esophagitis |  |  |  |  |
| High neighborhood SES / ADI 1-15 | 1.00 (Ref) | 1.00 (Ref) | 1.00 (Ref) | 1.00 (Ref) |
| Middle neighborhood SES / ADI 16-85 | 1.04 (1.01, 1.07) | 1.01 (0.98, 1.04) | 1.01 (0.98, 1.04) | 1.02 (0.99, 1.05) |
| Low neighborhood SES / ADI 86-100 | 1.11 (1.07, 1.16) | 1.02 (0.98, 1.06) | 1.02 (0.98, 1.06) | 1.03 (0.98, 1.07) |
|  |  |  |  |  |
| Inflammatory bowel disease |  |  |  |  |
| High neighborhood SES / ADI 1-15 | 1.00 (Ref) | 1.00 (Ref) | 1.00 (Ref) | 1.00 (Ref) |
| Middle neighborhood SES / ADI 16-85 | 1.07 (0.95, 1.20) | 1.06 (0.94, 1.19) | 1.00 (0.89, 1.14) | 1.00 (0.89, 1.14) |
| Low neighborhood SES / ADI 86-100 | 1.09 (0.93, 1.28) | 1.01 (0.85, 1.19) | 0.94 (0.78, 1.12) | 0.93 (0.78, 1.12) |
|  |  |  |  |  |
| Peptic ulcer disease |  |  |  |  |
| High neighborhood SES / ADI 1-15 | 1.00 (Ref) | 1.00 (Ref) | 1.00 (Ref) | 1.00 (Ref) |
| Middle neighborhood SES / ADI 16-85 | 1.08 (0.99, 1.18) | 1.07 (0.98, 1.17) | 1.06 (0.97, 1.16) | 1.07 (0.98, 1.17) |
| Low neighborhood SES / ADI 86-100 | 1.23 (1.11, 1.38) | 1.14 (1.01, 1.28) | 1.12 (0.99, 1.27) | 1.13 (1.00, 1.28) |
|  |  |  |  |  |
| Biliary tract disorders |  |  |  |  |
| High neighborhood SES / ADI 1-15 | 1.00 (Ref) | 1.00 (Ref) | 1.00 (Ref) | 1.00 (Ref) |
| Middle neighborhood SES / ADI 16-85 | 0.93 (0.89, 0.99) | 0.93 (0.88, 0.98) | 0.93 (0.88, 0.98) | 0.93 (0.88, 0.98) |
| Low neighborhood SES / ADI 86-100 | 0.97 (0.90, 1.04) | 0.93 (0.87, 1.01) | 0.93 (0.86, 1.01) | 0.93 (0.86, 1.01) |

ADI = Area Deprivation Index; CI = Confidence interval; OR = Odds ratio

Model 1 covariate included ADI restricted cubic spline terms only. Model 2 added covariates for age, sex, race/ethnicity, year of admission, end-stage renal disease status, and comorbid conditions. Model 3 added covariates for residence in a rural area, number of primary care providers per 100,000 persons, total number of specialists per 100,000 persons, hospital beds per 10,000 persons, and distance to the nearest hospital in miles. Model 4 added covariates for number of beds of admitting hospital, teaching status of admitting hospital, and public vs. private ownership of admitting hospital.
